# Shaken Identities, Reserve Masculine Capital, and the Lived Experiences of Aging Men with Breast Cancer

**DOI:** 10.1093/geroni/igab046.2583

**Published:** 2021-12-17

**Authors:** Edward Thompson, Renee Beard

**Affiliations:** 1 College of the Holy Cross, Broadview Heights, Ohio, United States; 2 College of the Holy Cross, College of the Holy Cross, Massachusetts, United States

## Abstract

A question that begs to be examined is: How does aging men’s discovery they have breasts as a result of their breast cancer diagnosis and having a breast removed through a mastectomy, affect their masculine subjectivities and practices, as they also go about also living with a life-threatening illness? The present study aimed to better understand how men come to live with the knowledge that they have both breasts and cancer. Interviews with seventeen men in the U.S. (mean age 62.8) with a breast cancer diagnosis, mastectomy, and, most often, post-surgical hormonal treatment uncovered stories of body-self disruption and identity dilemmas. All the men’s identities had been shaken. After their mastectomy, they were reminded every morning that the body reflected in the mirror differed significantly from who they once were. Their stories revealed strategic themes: how they lived with cancer by slightly modifying conventional masculinities; and how others interacted with them, with the exception of mammography technologists, in terms of their gender, not their atypical illness. Only a few men initially felt their breast cancer was a gendered stigma. Noticeable was how the historical era when diagnosed and the age of the man at diagnosis contextualized their illness stories. In this presentation, three cases are used to exemplify the men’s varied experiences with their non-normative bodies and their commonality in finding reserves of masculine capital to rebuke the existential loneliness of a man with breast cancer.

